# *Rec-8* dimorphism affects longevity, stress resistance and X-chromosome nondisjunction in *C. elegans*, and replicative lifespan in *S. cerevisiae*

**DOI:** 10.3389/fgene.2014.00211

**Published:** 2014-08-04

**Authors:** Srinivas Ayyadevara, Çagdas Tazearslan, Ramani Alla, James C. Jiang, S. Michal Jazwinski, Robert J. Shmookler Reis

**Affiliations:** ^1^Central Arkansas Veterans Healthcare System, VA Medical CenterLittle Rock, AR, USA; ^2^Department of Geriatrics, University of Arkansas for Medical SciencesLittle Rock, AR, USA; ^3^Department of Biochemistry and Molecular Biology, University of Arkansas for Medical SciencesLittle Rock, AR, USA; ^4^Tulane Center for Aging and Department of Medicine, Tulane University Health Sciences CenterNew Orleans, LA, USA

**Keywords:** longevity, lifespan, stress resistance, *C. elegans*, *S. cerevisiae*, genetics of aging, *rec-8* or *REC8*, meiotic cohesin

## Abstract

A quantitative trait locus (QTL) in the nematode *C. elegans*, “*lsq4*,” was recently implicated by mapping longevity genes. QTLs for lifespan and three stress-resistance traits coincided within a span of <300 kbp, later narrowed to <200 kbp. A single gene in this interval is now shown to modulate all *lsq4*-associated traits. Full-genome analysis of transcript levels indicates that *lsq4* contains a dimorphic gene governing the expression of many sperm-specific genes, suggesting an effect on spermatogenesis. Quantitative analysis of allele-specific transcripts encoded within the *lsq4* interval revealed significant, 2- to 15-fold expression differences for 10 of 33 genes. Fourteen “dual-candidate” genes, implicated by both position and expression, were tested for RNA-interference effects on QTL-linked traits. In a strain carrying the shorter-lived allele, knockdown of *rec-8* (encoding a meiotic cohesin) reduced its transcripts 4-fold, to a level similar to the longer-lived strain, while extending lifespan 25–26%, whether begun before fertilization or at maturity. The short-lived *lsq4* allele also conferred sensitivity to oxidative and thermal stresses, and lower male frequency (reflecting X-chromosome non-disjunction), traits reversed uniquely by *rec-8* knockdown. A strain bearing the longer-lived *lsq4* allele, differing from the short-lived strain at <0.3% of its genome, derived no lifespan or stress-survival benefit from *rec-8* knockdown. We consider two possible explanations: high *rec-8* expression may include increased “leaky” expression in mitotic cells, leading to deleterious destabilization of somatic genomes; or REC-8 may act entirely in germ-line meiotic cells to reduce aberrations such as non-disjunction, thereby blunting a stress-resistance response mediated by innate immunity. Replicative lifespan was extended 20% in haploid *S. cerevisiae* (BY4741) by deletion of *REC8*, orthologous to nematode *rec-8*, implying that REC8 disruption of mitotic-cell survival is widespread, exemplifying antagonistic pleiotropy (opposing effects on lifespan vs. reproduction), and/or balancing selection wherein genomic disruption increases genetic variation under harsh conditions.

## Introduction

*C. elegans* was the first complex animal (metazoan) in which genes that can mediate evolutionary changes in lifespan, through natural selection, were enumerated and characterized by linkage mapping (Ebert et al., 1993, 1996). At least 11 quantitative trait loci (QTLs) that modulate lifespan in *C. elegans* were defined by studying the progeny of several independent inter-strain crosses, most of which were identified in multiple crosses (Ebert et al., 1993, 1996; Ayyadevara et al., 2001, 2003; Shmookler Reis et al., 2007). One such QTL, *lsq4* on chromosome IV, appeared in three different inter-strain crosses as highly significant linkage-mapping peaks for longevity (each *P* < 10^−9^) (Ebert et al., 1993, 1996; Ayyadevara et al., 2001, 2003; Shmookler Reis et al., 2007). Recombinant-congenic lines were identified and mapped, allowing the limits of this QTL to be defined absolutely rather than stochastically (as likelihood maxima). After the first round of recombination screening, the interval for the longevity QTL spanned 0.3 Mb, coinciding with QTL-mapping intervals that govern resistance to several stresses: acute temperature elevation, paraquat and ultraviolet irradiation (Vertino et al., 2011). A second round of recombinant screening narrowed the *lsq4* interval to <0.20 Mb (Vertino et al., 2011), providing a manageable set of positional candidate genes to test for functional consequences of altered expression.

Further experiments, reported here, have led to identification of the gene responsible for *lsq4*-linked traits. Two strains representing diverged alleles of this QTL, differing only within a region that comprises ~0.3% of the genome (0.3 Mb), were first compared by transcriptional profiling on microarrays, either considering all genes (“genomewide” analysis) or restricting our attention to just those genes that fall within the implicated QTL interval. The genome-wide survey defined the underlying gene as one involved in spermatogenesis or sperm-specific expression. Results of the interval-restricted survey guided more quantitative comparisons by real-time PCR, which implicated 10 genes as differential-expression candidates. Finally, candidate and control genes were tested by RNA interference to ask whether knockdown of their expression in the shorter-lived, stress-susceptible strain could recapitulate any of the phenotypes associated with the more-robust QTL allele. These studies led to discovery of a gene for which reduction of transcript levels below a threshold is both necessary and sufficient to account for all observed functional consequences of QTL dimorphism. We note that this endpoint has only rarely been attained through pursuit of linkage mapping for a complex trait (Shmookler Reis, 2003; Parsons et al., 2005; Lai et al., 2007; Shmookler Reis et al., 2007; Vertino et al., 2011; Stumpferl et al., 2012).

Most of the genes and pathways currently considered to regulate longevity were initially discovered through mutagenesis screens, and were replicated in other species by targeted knockout or occasionally by introduction of a dominant-negative or overexpressing transgene. Many other genes and pathways were first implicated by RNA-interference screens, but share the weakness of mutational screens: both are limited to targets that extend lifespan when disrupted, absent or under-expressed. Studies that begin with discovery by gene mapping complement such screens, because QTL detection is inherently unbiased as to whether a gene (or a given allele) favors or opposes longevity. It is limited only by the evolutionary requirement that at least two common alleles (natural variants) affecting the trait must exist in wild-derived populations, implying that neither ensures a selective advantage across a range of environments. Given this fundamental difference in our approach from the prevalent sources of discovery for longevity genes, it is not surprising that *rec-8* was previously unrecognized as a contributor to lifespan pathways, despite its present implication in longevity of taxa as diverse as nematodes, fungi and whales.

## Materials and methods

### Nematode strains

*C. elegans* strain CL2a (DR1345) is a wild-type strain isolated in California in 1972; we obtained DR1345 from the Caenorhabditis Genetics Center (St. Paul, MN). SR708 is a recombinant-inbred strain described in Vertino et al. (2011), derived from F2 progeny of an interstrain cross between DR1345 and Bergerac-BO (RW7000), followed by recurrent outcrossing to CL2a/DR1345 with marker-based selection for retention of the BO allele at *lsq4*.

### General methods

All worms were grown on plates of 1.7% agar in Nematode Growth Medium, “NGM” (Sulston and Hodgkin, 1988), at 20°C unless otherwise noted. Plates were spotted with a freshly grown central lawn of *E. coli* (Sulston and Hodgkin, 1988), either strain OP50, a leaky auxotroph for uracil, or strain HT115 for RNAi experiments. Other procedures are described in Vertino et al. (2011).

### Transcript quantitation

Gene expression at the transcript level was assessed in two ways—using full-genome microarrays and by real-time, reverse-transcription PCR (RT-PCR). cDNAs were reverse transcribed from total RNA preparations (RNeasy Mini kit, Qiagen), labeled by 5′ attachment of fluors (GeniSphere HS900), and hybridized (ArrayBooster, The Gel Company) to 22,490-feature, 60-mer oligonucleotide arrays printed on epoxy slides (Washington University Genome Sequencing Center, funded by NHGRI and HHMI). Slides were scanned on a ScanArray 5000 (Perkin-Elmer), data were normalized by array-median values, and analyzed using Significance Analysis of Microarrays [ver. 2.23B, Stanford University (Tusher et al., 2001)]. Quantitative RT-PCR was conducted on an MJ Research Opticon2 Thermal Cycler, using SYBR Green PCR Master Mix (Applied Biosystems) and normalizing transcript levels to the mean of three genes that did not vary significantly between strains (β-actin, T08G5.3, and Y71D11.3) (Ayyadevara et al., 2009).

### RNA interference and functional assays

Nematodes were picked at the L4 larval stage, and transferred to plates seeded with dsRNA-expressing bacteria [*E. coli* strain HT115 (DE3), transformed with expression plasmid L4440] and induced with IPTG, as previously described (Ayyadevara et al., 2009; Tazearslan et al., 2009). Progeny of those worms were maintained on the same dsRNA-expressing bacterial strain; or, where indicated, worms were studied after transfer at the L4/adult molt to plates seeded with dsRNA-expressing bacteria, on which they were subsequently maintained.

Longevity survivals were performed as described (Vertino et al., 2011), continuing dsRNA exposure throughout life. Worms, initially in groups of 35, were transferred every second day onto agar plates with fresh lawns of dsRNA-expressing bacteria, and scored for spontaneous motility, provoked movement, and pharyngeal pumping; worms displaying none of these traits were considered dead. For paraquat-resistance assays (Shmookler Reis et al., 2007), groups of 30 worms were maintained with RNAi exposure for 3.5 days, and transferred (as day-3 adults) to liquid survival medium containing 150 mM paraquat for variable periods of exposure, or to 0–80 mM paraquat at 20°C for 3 days (replacing paraquat medium daily). Deaths were assessed as above, at the times indicated. For thermotolerance assays, modified from Lithgow et al. (1995), groups of 30 worms were picked 24 h after the L4/adult molt onto 6-cm agar plates and immersed to a depth of 0.5 cm in a 35.5°C water bath. Plates were removed at the indicated times and worms were scored for survival in the same sequence. Male counts were performed 1 day after the L4/adult molt, for worms whose parents and grandparents (prior to egg-laying) had been exposed to dsRNA-expressing bacteria.

### Replicative lifespan assays in *saccharomyces cerevisiae*

Replicative lifespans were determined for budding yeast as described previously (Jiang et al., 2000). Briefly, YPK9 and BY4741 cells were grown in liquid YPG medium (2% peptone, 1% yeast extract, 3% glycerol) to eliminate petites unable to utilize the non-fermentable carbon source. They were then spotted on YPD plates (2% peptone, 1% yeast extract, 2% glucose, 2% agar). Budding cells were selected by micromanipulation and arranged in a row in an isolated region of the plate. The new buds were dissected to initiate the experiment, and the mother cells were discarded. The plates were incubated at 30°C and periodically examined under the microscope. The lifespan of a cell was recorded as the number of buds it produced (consecutively removed by micromanipulation and discarded), before it ceased dividing and lost refractility. Data are summarized by mean and standard deviation of the maximum generation (bud) number achieved in each of 40 mother cells.

### Statistics

Transcript levels ascertained on microarrays were considered to display differential expression if their genome-wide false discovery rate *q* (the expected frequency of false positives for the entire microarray) was less than 5% (Tusher et al., 2001); in order to further restrict the gene list, a lower threshold (e.g., *q* < 1% or *q* = 0) was sometimes used. A separate cohort was expanded and its RNA extracted for each microarray, so that the biological *N* was equal to the number of assays.

In considering the significance of quantitative intergroup differences (e.g., for RT-PCR data), two strains were usually compared by two-tailed heteroscedastic *t*-tests (appropriate to samples of unknown or unequal variance), considering *N* to be the number of biological preparations used for each group (i.e., ignoring technical replicates). *P*-values less than 0.05 were considered to have attained nominal statistical significance despite multiple comparisons, so as not to inflate the Type II error rate in low-power assays (e.g., utilizing 2–4 biological samples of each strain).

In all *C. elegans* survival comparisons, significance was assessed by Cox-Mantel *F*-test, a conservative non-parametric statistic (Lee and Wang, 2003); similar *P*-values were obtained by the Gehans-Wilcoxon log-rank test. Comparisons of nematode proportions surviving in a dose-response experiment utilized the chi-squared test for each dose. Yeast replicative lifespan differences were evaluated by Mann–Whitney–Wilcoxon rank-sum tests.

## Results

### Effects of QTL alleles on genome-wide transcript levels

We grew four independent cohorts each of strains CL2a and SR708, a near-congenic line differing from CL2a only by a Bergerac-BO-derived segment on chromosome 4, which contains the *lsq4* QTL (Vertino et al., 2011) between **snp_Y45F10C** (13.33 Mb) and **snp_W02A2** (13.65 Mb). Four microarrays (Washington Univ. Genome Sequencing Center) were hybridized with dual-label cDNA probes, reverse-transcribed from the RNA preparations obtained from these cohorts. Despite the very narrow *lsq4*-spanning region of Bergerac-BO DNA substituted in the CL2a genome to create SR708 (0.32 Mb, containing <50 genes), more than 150 genes were differentially expressed between these strains, at a false discovery rate (*q*) of ≤1%; a substantial majority were expressed at a lower level in SR708 than in CL2a. The 22 most significantly differential genes, those with *q* = 0, are listed in Table 1A. Of those genes, 12 are *msp* genes encoding Major Sperm Proteins, and another 3 encode proteins with MSP-like domains. The remaining 7 genes include a basic sperm nuclear protein unrelated to MSPs, two proteins with eggshell-protein domains, and two of the 12 NSPA-family members. The MSP family comprises >50 small basic proteins implicated in extracellular signaling and cytoskeletal functions, many of which are enriched in the amoeboid sperm cells. We used RT-PCR targeting two of these genes, *msp-142* and *msp-56*, to confirm their differential expression as indicated on microarrays (Table 1B). To assess the generality of *lsq4* allelic effects on *msp* expression, we also quantified transcripts for a conserved *msp* region (Table 1B). Overall, *msp* expression based on RT-PCR differed by nearly 5-fold between *lsq4* alleles, a less extreme ratio than observed on microarrays.

**Table 1:** **Genes with highly differential expression dependent on the *lsq4* allele**.

**Gene name**	**Gene ID**	**CL2a/SR708**	Motifs^*^	**Gene/functions of encoded protein**
**(A)**				
*msp-142*	K05F1.2	54.4	A, C_2_	*msp-142* encodes a member of the Major Sperm Protein (MSP) family of >50 small basic proteins involved in extracellular signaling and cytoskeletal functions
*msp-63*	K05F1.7	59.1	C_2_	*msp-63* encodes a member of the Major Sperm Protein (MSP) family
*msp-56*	K07F5.3	17.2	A	*msp-56* encodes a member of the Major Sperm Protein (MSP) family
*msp-38*	K08F4.8	21.4	–	*msp-38* encodes a member of the Major Sperm Protein (MSP) family
*msp-59*	ZK354.11	12.8	A	*msp-59* encodes a member of the Major Sperm Protein (MSP) family
*msp-51*	ZK354.5	9.7	A, C	*msp-51* encodes a member of the Major Sperm Protein (MSP) family
*msp-49*	C34F11.6	19.2	A_4_	*msp-49* encodes a member of the Major Sperm Protein (MSP) family
*msp-78*	T13F2.11^**^	55.8	C	*msp-78* encodes a member of the Major Sperm Protein (MSP) family
Unknown protein	F58A6.9	24.2	A_2_	Similar to Pfam domain PF00635 and to Interpro IPR000535 (Major Sperm Protein)
*msp-142*	K05F1.2	203.3	A, C_2_	(Second oligonucleotide specific to this gene in *C. elegans* microarrays)
*nspa-5*	ZC412.6	367.3	A	Nematode Specific Peptide family/group A (12 genes)
*msp-32*	R05F9.3	236.3	A	*msp-32* encodes a member of the Major Sperm Protein (MSP) family
Unknown protein	T27A3.4^**^	55.4	C_7_	Similar to *S. solidissima* sperm nuclear basic protein PL-I isoform PLIa
*msp-10*	K07F5.2	14.0	–	*msp-10* encodes a member of the Major Sperm Protein (MSP) family
Unknown protein	K07F5.5	>500	A_19_	Contains a domain similar to Interpro domain IPR002952 (Eggshell protein)
*msp-50*	C34F11.4	>500	A	*msp-50* encodes a member of the Major Sperm Protein (MSP) family
*ssp-11*	T28H11.6^*^	>500	A	Sperm-specific family class P, contains major sperm protein (MSP) domain
*nspa-8*	W06A7.5	>500	–	Nematode Specific Peptide family/group A (comprising 12 genes)
Unknown protein	C35D10.11^*^	81.5	C	Uncharacterized protein, contains major sperm protein (MSP) domain
Unknown protein	F15E11.1	8.3	–	F15E11.1 encodes a 17.4 kDa protein (CE16999) of unknown function that is 7-fold more abundant in glp-1 mutants (which lack germline) than in WT
Unknown protein	F41F3.3	16.4	–	Contains domain similar to IPR002952 (Eggshell protein), and IPR000694 (Proline-rich region)
*col-3*	T28C6.6	2.7	–	*col-3* encodes a collagen (types IV, XIII) that affects body morphogenesis, larval viability, and locomotion; expressed during all developmental stages
**(B)**				
**RT-PCR primers targeting:**		**CL2a/SR708**		**Significance (by two-tailed *t*-test, unequal variance)**
MSP-conserved region		4.7		*p* < 0.04
*msp-56*		4.1		*p* < 0.07
*msp-142*		4.4		*p* < 0.05

**(A)** Microarray results for genes with q (SAM False Discovery Rate) ≈0, for analysis of 4 dual-fluor slides (Genome Sequencing Center, Washington University). **(B)** Confirmation by Real-Time Polymerase Chain Reaction, in which cDNAs reverse-transcribed from 4 RNA preparations per strain (SR708 and CL2a) were amplified with primer pairs targeting a conserved region shared by many msp genes, or regions specific to msp-56 and msp-142. Motifs in 1-kbp regions upstream of listed genes: “A,” rraAACGGGACrwc; “C,” akCATATATArc; subscripts reflect multiple occurrences.

* Orthologous to, or

** adjoining an ortholog of, a male-sperm-specific Ascaris suum MSP-domain protein (Tarr and Scott, 2004).

In view of the striking enrichment observed for structurally or functionally related genes, and considering that none of the top 50 genes are located within the *lsq4* interval, we inspected their upstream 1-kb regions with modules of RSAT (regulatory sequence analysis tools, http://rsat.ulb.ac.be/rsat/) to seek shared motifs. “Oligo-analysis” identified 3 over-represented motifs: AACGGGAC (enriched 32-fold, *P* < 2E−12), CATATATA (10-fold, *P* < 5E−7) and AAAGTTTC (4-fold, *P* < 2E−5). “Matrix scan” found extended versions of the first two motifs, rraAACGGGACrwc (*P* < 3E−33) and akCATATATArc (*P* < 2E-6). Although such enrichments were highly significant, *lsq4*-allele-specific binding of one or more transcription factors to these motifs could not account for the differential transcript abundances observed (see “Motifs” column, Table 1).

Differential-expression patterns can provide valuable clues as to the nature of the underlying gene that is dimorphic between QTL alleles. For example, altered expression of sperm-specific genes might reflect a change in the frequency of males, since male *C. elegans* not only produce more sperm than do hermaphrodites (>1000 vs. ~300 per worm), but also produce sperm that differ in many of their constituent proteins from hermaphroditic sperm (Tarr and Scott, 2004). On assessing the frequency of males, we noted a distinct “HIM” (**h**igh **i**ncidence of **m**ales) trait in strain CL2a/DR1345 [1.2 ± 0.2 (SEM) %, 1.4 ± 0.4% in two experiments, total *N* = 4100], well above the 0.1–0.2% seen in most other wild-type strains such as Bristol-N2, Bergerac-BO [RW7000], or a different CL2a subline [CB4857] (Hodgkin and Doniach, 1997). This trait was reverted in SR708, for which the male incidence was 0.2 ± 0.1% (total *N* = 4750), 6- to 7-fold less than CL2a (chi-squared *P* < 10^−5^ in each experiment), whereas none of the longer-lived recombinant-congenic lines (described in Vertino et al., 2011) differed from CL2a. Male incidence thus constitutes another allele-specific trait of *lsq4*, in addition to longevity, thermotolerance, and paraquat resistance (Vertino et al., 2011).

### QTL allele effects on transcript abundances for *lsq4* positional-candidate genes

We then restricted our expression survey to genes in the *lsq4* interval (Table 2), by selecting data from the full-genome microarrays (see previous section). We identified 18 genes in the *lsq4* interval, for which transcript levels differed between CL2a and the SR708 line, and retested these by quantitative real-time reverse-transcriptase PCR (qRT-PCR). Considering only the biological *N* of 2 (two independent grow-ups of each strain, distinct from those initially screened in microarrays), and not the technical duplication of assays for each of those, 10 of the 18 genes assessed showed allele-dependent changes that were nominally significant (*P* ≤ 0.05), whereas no more than one would be expected by chance (Table 3).

**Table 2:** **Genes in the *lsq4* interval—positional candidates for life-span determinants**.

**Gene: protein product**	**Chromosome-4 position**	**YAC clone**
*far-6*: fatty-acid/retinol binding protein	13328335-13329295	W02A2.2
*pqn-74*: an unknown protein with a prion-like (Q/N-rich) domain	13331364-13329512	W02A2.3
Gene encoding an unknown, uncharacterized protein	13342834-13346165	W02A2.5
*rec-8*: cohesin protein involved in meiotic recombination	13353169-13349096	W02A2.6
*mex-5*: dual-zinc finger protein, C ×8 C ×5 C ×3 H type	13353692-13356087	W02A2.7
*srsx-25*: 7-transmembrane receptor (rhodopsin family)	13365145-13368357	Y62E10A.4
*agt-1*: ortholog of O6-alkylguanine DNA alkyl/methyltransferase	13369396-13368435	Y62E10A.5
*rla-2*/*rpa-2*: ribosomal protein, large subunit, acidic	13370269-13369400	Y62E10A.1
Unnamed ortholog of Pop7, subunit shared by RNAses P, MRP	13370629-13371589	Y62E10A.2
Ferredoxin/adrenodoxin oxidoreductase, mitoch. inner membr.	13371972-13377345	Y62E10A.6
*mau-8*: phosducin-like protein PhLP, electron transporter	13377990-13380453	Y62E10A.8
*rab-19*: Ras family (contains ATP/GTP-binding P loop)	13384292-13382037	Y62E10A.9
*mdt-9*: Mediator protein; RNAi has deleterious phenotypes	13386270-13387337	Y62E10A.11
*lsm-3*: small nuclear ribonucleoprotein (snRNP), Sm-like	13387179-13389575	Y62E10A.12
Phosphoserine phosphatase homolog	13398775-13389133	Y62E10A.13
Uncharacterized protein; mutant, RNAi affect embryogenesis	13400489-13399043	Y62E10A.14
*cyp31A5*:cytochrome P450; RNAi alters developmt/metabolism	13401282-13403807	Y62E10A.15
*gcl-1*: hom. human germ-cell-less protein w BTB/POZ domain	13412873-13408989	Y62E10A.16
Y62E10A.17 encodes an essential AP-2 transcription factor	13413541-13415930	Y62E10A.17
*pik-1*: eukaryotic Ser/Thr protein kinase, homol. to pelle/IRAK	13416768-13421501	K09B11.1
*nol-9*: partial hom. human nucleolar prot. 9; ATP/GTP binding	13422474-13429350	K09B11.2
Tyr protein kinases, homologous to human/mouse FER	13434044-13431218	K09B11.5
*mam-3*: Meprin-, A5-protein-, PTPmu-domain protein	13434218-13445285	K09B11.10
U6 snRNAs U6 8, -9, -10, -11, -12, and -13 (uncharacterized)	13435761-13443822	K09B11.11–16
*uso-1*: orthol. of vesicle tethering protein, ER-to-golgi transport	13454774-13446008	K09B11.9
Zinc finger (C2H2–5); homologous to Fez TF, ZNF195/p57KIP2	13466115-13469378	Y38H8A.5
Ser/Thr protein kinase, related to yeast AMPK of Snf4 complex	13472239-13470538	Y38H8A.4
Ser/Thr/Tyr protein kinase, homol. human tau tubulin kinase 2	13472482-13473955	Y38H8A.3
Homolog of Pleckstrin, winged-helix repressor, and glutaredoxin	13492547-13497117	Y45F10A.7
*tbc-9*: protein w. RabGAP/TBC and calcium-binding domains	13502828-13476911	Y45F10A.6
*nlp 17*: neuropeptide-like protein	13505734-13505243	Y45F10A.5
*seld-1*: AIR-synthase-related protein; selenophosphate synthase	13506055-13509191	Y45F10A.4
*puf-3*: Pumilio family RNA-binding translational repressor	13512144-13510282	Y45F10A.2
*dcap-2*: mRNA decapping enzyme, DCP2 pyrophosphatase	13521848-13513345	F52G2.1

^*^Physical locations reflect WormBase Release WS233.

**Table 3:** **Genes in the *lsq4* interval for which transcript level depends on the *lsq4* allele**.

	**(A) Gene product**	**(B) Clone name**	(C) Transcript ratio CL2a/SR708* (mean Δ cycle no. ± *SD*)	**(D) *P*-value**
Expression higher in CL2a	PQN-74 protein, Q/N-rich (prion) domain	W02A2.3	15.0 (3.8 ± 1.0)	0.04
	S/T/Y kinase, tau tubulin kinase 2 homolog	Y38H8A.3	5.6 (2.5 ± 0.3)	0.05
	Tyrosine kinase, catalytic subunit	K09B11.5	5.2 (2.4 ± 0.4)	0.03
Expression higher in SR708	FAR-6 (fatty-acid/retinol binding protein)	W02A2.2	0.52 (−0.9 ± 0.1)	0.01
	AGT-1, DNA O6-alkyl-G alkyltransferase	Y62E10A.5	0.44 (−1.2 ± 0.2)	0.04
	REC-8, meiosis-specific cohesin subunit	W02A2.6	0.34 (−1.6 ± 0.2)	0.03
	LSM-3 small nuclear ribonucleoprotein	Y62E10A.12	0.31 (−1.7 ± 0.2)	0.03
	MEX-5 zinc-finger protein	W02A2.7	0.09 (−3.5 ± 0.3)	0.005
	Unchar. protein required in embryogenesis	Y62E10A.14	0.09 (−3.6 ± 0.2)	0.005
	PUF-3 Pumilio-family RNA-binding protein	Y45F10A.2	0.07 (−3.9 ± 0.3)	0.003

Genes showing significant allelic differences are listed in order of relative transcript abundance based on real-time PCR (column C), after initial screening with dual-fluor microarrays. Real-time PCR results show CL2a/SR708 transcript ratios determined from replicate biological samples, each assessed in duplicate for a total of 4 assays. Data in parentheses are cycle-number differences (mean ± SD, SR708 –CL2a) to reach an amplification threshold. P-values (column D), prior to Bonferroni adjustment, are for Behrens-Fisher 2-tailed t-tests appropriate to samples of unknown or unequal variance, considering two biological samples as the n per group.

### Functional assessment of *lsq4* candidate genes by RNA-interference knockdown

Although QTLs have their origins in DNA-sequence polymorphisms that could either alter protein sequence and function or have *cis* effects on transcription (or more rarely, both), allelic shifts in transcript level are relatively common and their functional importance can be readily assessed by RNA interference (RNAi). The 10 *lsq4*-interval genes confirmed to be differentially expressed dependent on the *lsq4* allele present (listed in Table 3) were tested along with 4 control genes, to determine whether RNAi can shift the strain with higher transcript level toward the phenotypes of the lower-expression strain. Increased longevity in response to RNAi is a relatively exceptional outcome, reported for 1.2–1.4% of genes surveyed in high-throughput, single-point assays (Hamilton et al., 2005; Hansen et al., 2005), and thus permits stronger inference than the far more common result of reduced survival.

CL2a or SR708 worms were fed for 3 days beginning at the L4/adult molt, on bacteria expressing gene-specific double-stranded RNA (dsRNA) constructs from the Ahringer library (Kamath and Ahringer, 2003) if they were present there. We designed dsRNA-expression inserts for selected genes that were not represented in the Ahringer library, which were synthesized (IDT, San Diego CA), ligated into an expression plasmid, and transfected into bacteria, as reported previously (Ayyadevara et al., 2007, 2009). In the case of *rec-8*, encoding a cohesion protein (“cohesin”) required for alignment of homologous and sister chromatids during meiosis, we observed ~4-fold (73, 76% in two assays) suppression relative to worms fed the empty expression vector or an unrelated dsRNA; the range for all tested genes was 2- to 28-fold suppression (data not shown). We then assessed the treated worms for RNAi effects on established *lsq4* traits: longevity, paraquat resistance, thermotolerance, and male frequency.

#### Longevity

**Figure 1:**
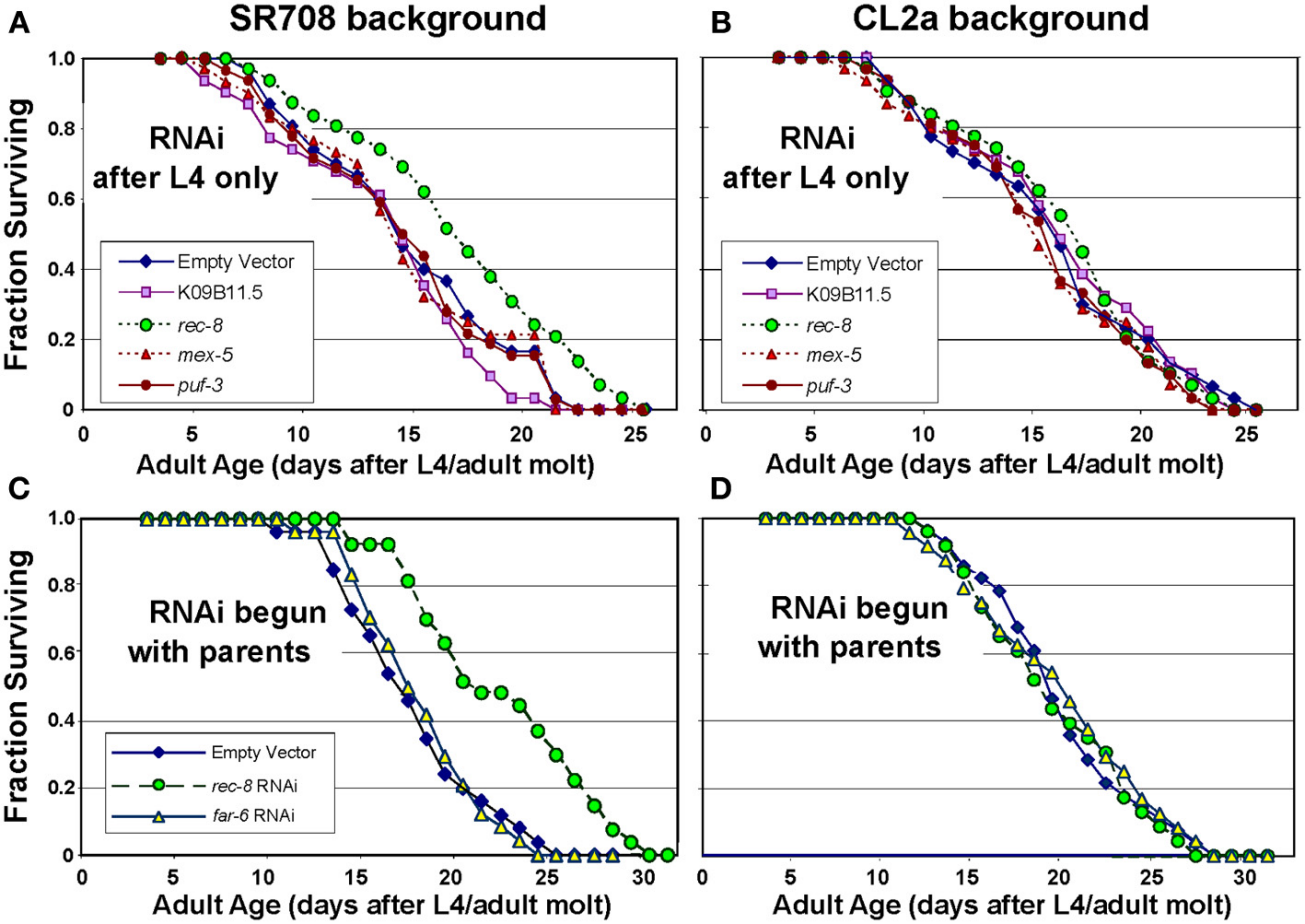
***Rec-8* knockdown extends lifespan, dependent on genetic background in the *lsq4* region**. Groups of 35 worms were fed on bacteria induced to express the indicated dsRNA (or empty expression vector) continuously from the L4/adult molt **(A,B)** to avoid possible developmental effects, or initiated with the L4/adult molt of the parents of tested worms **(C,D)** to maximize knockdown. Lifespan survivals were monitored as described (see Methods). Of the indicated genes targeted by dsRNAs, only K09B11.5 had been found to be expressed at higher levels in CL2a, the longer-lived strain, and thus predicted to possibly reduce CL2a longevity; the other 4 genes were expressed at higher levels in SR708, and thus were predicted to extend survival of treated SR708, if causal to the QTL effect on longevity. **(A,C)** Lifespan survivals of control vs. dsRNA-treated SR708 adults; **(B,D)** Lifespan survivals of control and dsRNA-treated CL2a adults.

In an initial screen for effects of RNA interference on lifespan, genes in the *lsq4* interval were selected for RNAi suppression of expression in either parental strain CL2a or the recombinant line SR708 (differing from CL2a by insertion of the Bergerac-BO-derived *lsq4* allele); positive or suggestive results were repeated multiple times (Table 4). Significant lifespan extension (*P* < 0.0006 to *P* < 4 × 10^−5^ prior to correction for multiple measures) was seen only for RNA interference targeting *rec-8*, and only in the SR708 line. Table 4 summarizes data from three independent survivals, in which *rec-8* dsRNA treatment increased the longevity of SR708 worms to roughly the same level as CL2a (with or without dsRNA). Two of these survival experiments are illustrated in Figure 1. In Figure 1A, mean adult lifespan at 20°C was extended 26%, from 13.5 ± 0.7 d (mean ± s.e.m.) for SR708 adults fed bacteria carrying an empty dsRNA expression vector, to 17.0 ± 0.9 d for those exposed to *rec-8* dsRNA (Cox-Mantel *P* < 0.0004). Note that inclusion of the period of larval development would add 2.5 days to the adult lifespan for each strain, to obtain the “total lifespan” more commonly shown. In contrast, the mean adult longevity of strain CL2a, 16.2 ± 0.9 d, was not shifted significantly (<3% change) as a result of any dsRNA treatment (Figure 1B and Table 4). In this experiment, RNA interference began at the L4/adult molt to avoid embryonic lethality as reported previously (Pasierbek et al., 2001). A very similar life extension relative to empty-vector control (25%, *P* < 4 × 10^−5^), however, was obtained in a second experiment wherein dsRNA treatment began in the parental generation (Figures 1C,D, and Table 4) to maximize the impact of knockdown. This resulted in substantial but transient embryonic lethality (Pasierbek et al., 2001), but no subsequent impairment in worms that completed embryogenesis. A third experiment also confirmed significant life extension by *rec-8* (Table 4; *P* < 0.0006), but calculation of the mean age at death was complicated by censorship of worms lost due to burrowing in the agar. The other genes tested here (described briefly in Table 2 and Discussion) did not significantly affect longevity.

**Table 4:** **Survival data for near-isogenic strains SR708 and CL2a, fed on *E. coli* expressing the indicated dsRNAs**.

**Gene targeted by RNAi**	***n***	**Median survival [days]**	**Mean survival [days], (fold change)**	**Standard deviation, [s.e.m.]**	Significance of difference from control^*^
**(A) STRAIN SR708 (EXPERIMENT 1, RNAi INITIATED AT L4/ADULT MOLT)**					
None (empty vector)	35; 25	14.5	13.5 (1.00×)	3.7 [0.7]	–
K09B11.5	35; 30	14.5	13.5 (1.00×)	4.3 [0.8]	N.S.
*far-6*	35; 25	16.5	15.5 (1.15×)	3.2 [0.7]	N.S.
*rec-8*	35; 29	17.5	17.0 (**1.26×**)	5.1 [0.9]	*P* < 0.0004^*^
*mex-5*	35; 22	13.5	12.6 (0.93×)	3.6 [0.8]	N.S.
*puf-3*	35; 27	13.5	12.1 (0.90×)	3.7 [0.7]	N.S.
**(B) STRAIN CL2a (EXPERIMENT 1, RNAi INITIATED AT L4/ADULT MOLT)**					
None (empty vector)	35; 30	16.5	16.2 (1.00×)	4.9 [0.9]	–
K09B11.5	35; 22	16.5	16.4 (1.01×)	4.6 [0.9]	N.S.
*far-6*	35; 28	16.5	17.1 (1.06×)	4.7 [0.9]	N.S.
*rec-8*	35; 29	17.5	16.5 (1.02×)	4.6 [0.9]	N.S.
*mex-5*	35; 21	15.5	15.8 (0.98×)	4.7 [0.9]	N.S.
*puf-3*	35; 24	16.0	16.0 (0.99×)	4.5 [0.9]	N.S.
**(C) STRAIN SR708 (EXPERIMENT 2, RNAi INITIATED FOR PARENTS OF TESTED WORMS)**					
None (empty vector)	35; 24	17.5	18.0 (1.00×)	3.6 [0.7]	–
*far-6*	35; 24	18.0	18.2 (1.01×)	3.2 [0.7]	N.S.
*rec-8*	35; 27	21.5	22.5 (**1.25×**)	4.7 [0.9]	*P* < 0.00004^*^
**(D) STRAIN CL2a (EXPERIMENT 2, RNAi INITIATED FOR PARENTS OF TESTED WORMS)**					
None (empty vector)	35; 28	19.5	20.0 (1.00×)	4.2 [0.8]	–
*far-6*	35; 24	20.5	20.0 (1.00×)	4.9 [1.0]	N.S.
*rec-8*	35; 23	19.5	19.6 (0.98×)	4.4 [0.9]	N.S.
**(E) STRAIN SR708 (EXPERIMENT 3, RNAi INITIATED FOR PARENTS OF TESTED WORMS)**					
None (empty vector)	35; 19	16.5	15.6 (1.00×)	2.9 [0.7]	–
*far-6*	35; 14	18.0	17.9 (1.14×)	3.4 [0.9]	*P* ≈ 0.05^*^
*rec-8*	35; 30	18.5	18.0 (**1.16×**)	2.9 [0.5]	*P* < 0.0006^*^
**(F) STRAIN CL2a (EXPERIMENT 3, RNAi INITIATED FOR PARENTS OF TESTED WORMS)**					
None (empty vector)	35; 19	21.5	20.3 (1.00×)	3.2 [0.7]	–
*far-6*	35; 17	20.5	20.3 (1.00×)	2.2 [0.5]	N.S.
*rec-8*	35; 24	20.5	19.7 (0.97×)	2.5 [0.5]	N.S.

* Based on Cox-Mantel F-test, each experiment treated separately.

n, initial number; deaths. Fold change is bolded when differences from controls are significant.

#### Paraquat survival

**Figure 2:**
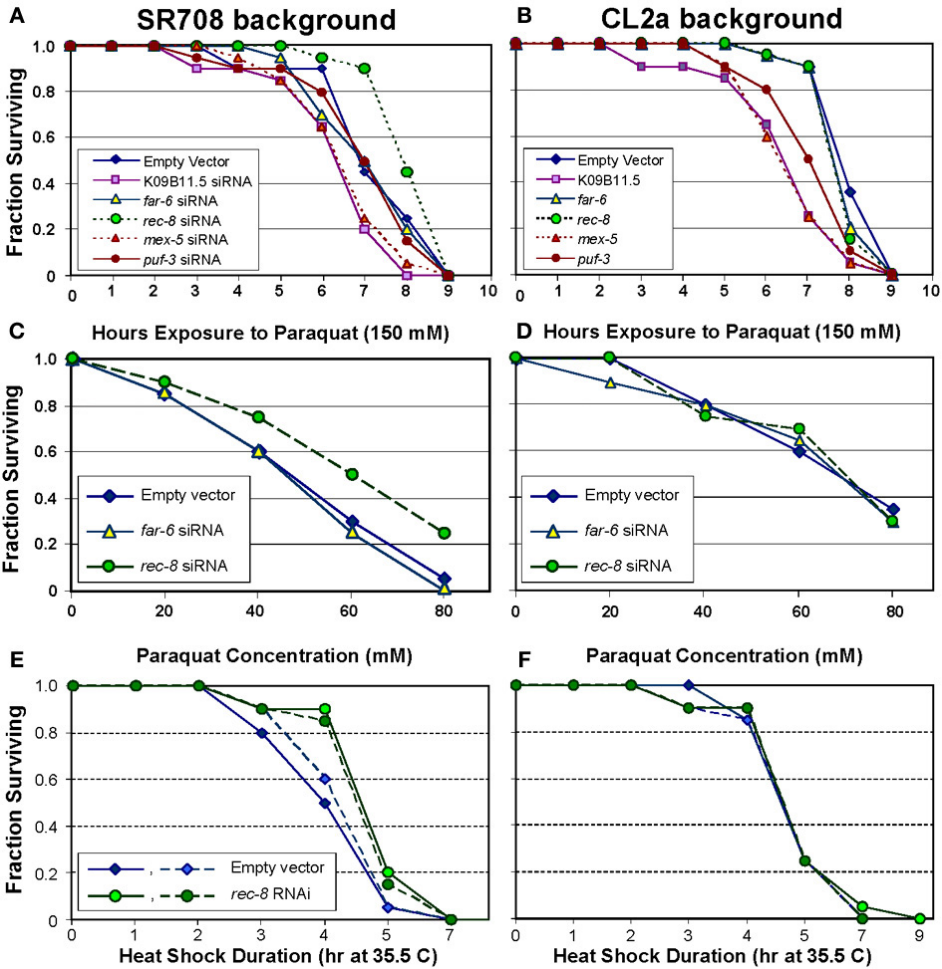
***Rec-8* knockdown improves resistance to oxidative and thermal stresses, dependent on the *lsq4*–region background**. Groups of 30 worms were fed on bacteria induced to express the indicated dsRNA (or empty expression vector) for 3 days from the L4/adult molt. They were then transferred to medium containing paraquat, and their subsequent survival monitored (see Methods). Of the indicated genes targeted by dsRNAs, only K09B11.5 had been found to be expressed at higher levels in CL2a, the longer-lived strain, and thus predicted to possibly reduce CL2a survival; the other 4 genes were expressed at higher levels in SR708, and thus were predicted to extend survival of treated SR708, if causal to the QTL effect on paraquat resistance. **(A,B)** Time courses of paraquat survival for control and dsRNA-treated adult worms, either **(A)** strain SR708, or **(B)** strain CL2a. **(C,D)**. Paraquat dose-response curves for control vs. dsRNA-treated adults of strain **(C)** strain SR708, or **(D)** strain CL2a. **(E**,**F)** Thermotolerance survivals following abrupt transfer of standard survival plates from 20 to 35.5°C, containing control or dsRNA-treated adults of **(E)** strain SR708, or **(F)** strain CL2a. Each panel for heat-shock survival shows combined results from two independent experiments (solid and dashed lines indicate experiments 1 and 2, respectively). All panels show data representative of 2–4 experiments of each type.

Paraquat exposure constitutes a toxic oxidative stress, through generation of superoxide radical (Ebert et al., 1993, 1996; Ayyadevara et al., 2001, 2003; Shmookler Reis et al., 2007). As illustrated in Figure 2A, siRNA targeting *rec-8* was the only RNA-interference treatment that improved paraquat survival for strain SR708. This dsRNA-expressing construct extended survival by 17%, to 8.5 ± 0.3 [s.e.m.] h (Figure 2A; *P* < 0.01 by log-rank test), very close to that of mock-treated CL2a (8.3 ± 0.2 h; Figure 2B). In CL2a worms, however, *rec-8* RNAi had no effect, as might be expected if the longer-lived *lsq4* allele already expressed REC-8 at levels near optimal for adult survival. Conversely, RNAi to K09B11.5 (encoding a tyrosine protein kinase) reduced paraquat survival in the CL2a strain (Figure 2B; 25% decrease, *P* < 6E–5), to a greater extent than in SR708 (Figure 2A; 16%, *P* < 0.04). Although these data are consistent with a survival-curtailing role for K09B11.5 in view of its 5-fold higher expression in CL2a, they offer only weak support for a causal role because RNA interference often impairs robustness, presumably by disruption of diverse pathways.

The *lsq4* allelic difference in paraquat resistance, and its reversal by RNAi targeting *rec-8*, were confirmed by dose-response experiments in which worms were monitored for survival in varying concentrations of paraquat. As illustrated in Figures 2C,D, RNAi to *rec-8* increased the paraquat LD_50_ by >30% (from 46 to 60 mM), for SR708 adults (chi-squared *P* < 0.005), but had no effect on CL2a.

#### Thermotolerance

Although *C. elegans* can tolerate a wide temperature range, sudden shifts of >14°C (termed “heat shock”) are lethal over the course of 3–7 h (Lithgow et al., 1995). We had observed an allelic difference in survival following abrupt transfer from 20 to 35.5°C, mapping coincidentally with the *lsq4* longevity trait (Vertino et al., 2011). SR708 worms, when grown on *E. coli* expressing *rec-8* dsRNA, acquired thermotolerance equivalent to CL2a (differing from SR708 only by the longer-lived *lsq4* allele), whereas control worms consuming only vector-bearing bacteria did not (Figure 2E). The same *rec-8* knockdown produced no improvement in survival of heat-shocked CL2a worms (Figure 2F). These plots (Figures 2E,F) show results of two independent experiments, indicated by solid vs. dashed lines, which were combined to assess significance of enhanced thermotolerance for SR708 worms fed *rec-8* RNAi (*P* < 0.002 by Cox-Mantel test).

#### Male incidence

**Figure 3:**
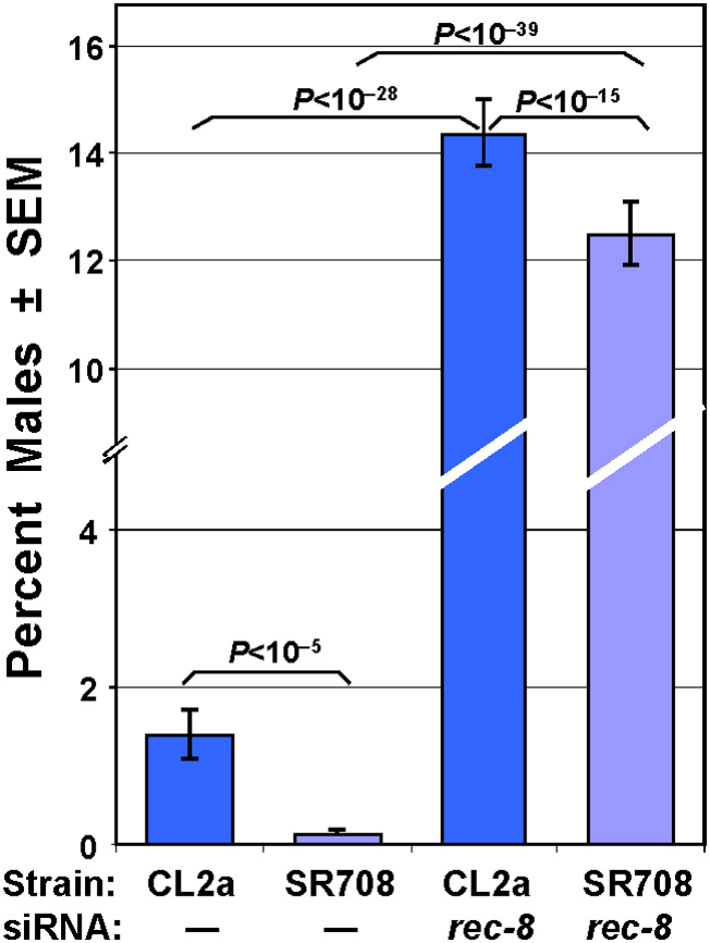
**Male frequency in the two *lsq4* isogenic strains**. The longer-lived *lsq4*-isogenic strain, CL2a, has a significant HIM (high incidence of males) phenotype (two “**–**” bars on left). RNA interference targeting *rec-8* greatly elevates the male frequency, but the difference between the two *lsq4*-allelic strains was preserved (two “***rec-8***” bars). *N* ranged from 1147–1920 progeny examined for male incidence per assessment. Data are shown for one of three experiments with similar results.

The longer-lived strain, CL2a, has a distinct HIM (**h**igh-**i**ncidence of **m**ales) trait with 1.8% males, whereas SR708 produced 0.2% males—a level typical of most wild-type strains (Figure 3). Continuous exposure of SR708 or CL2a hermaphrodites to *rec-8* dsRNA from the initiation of development (i.e., beginning in the prior generation to precede embryogenesis) raised the incidence of their male progeny to 12.5 and 14.3% respectively, close to the 15% level reported previously for either *rec-8* loss-of-function mutation or RNAi knock-down in an N2 background (Pasierbek et al., 2001). These high proportions of males greatly exceed those observed in SR708 or CL2a, whether untreated or exposed to a different dsRNA species, e.g., *far-6*. In several independent groups assessed together, for example, *rec-8* RNAi increased the male incidence of SR708 by 120- to 180-fold (*N* = 1267–1920 per group, each chi-squared *P* < 10^−39^), while in the CL2a strain male incidence rose 8- to 15-fold (*N* = 1147–1542 per group, each chi-squared *P* < 10^−28^). Very similar results were seen in repeat experiments.

### Deletion of *REC8* can extend the replicative lifespan of yeast

**Figure 4:**
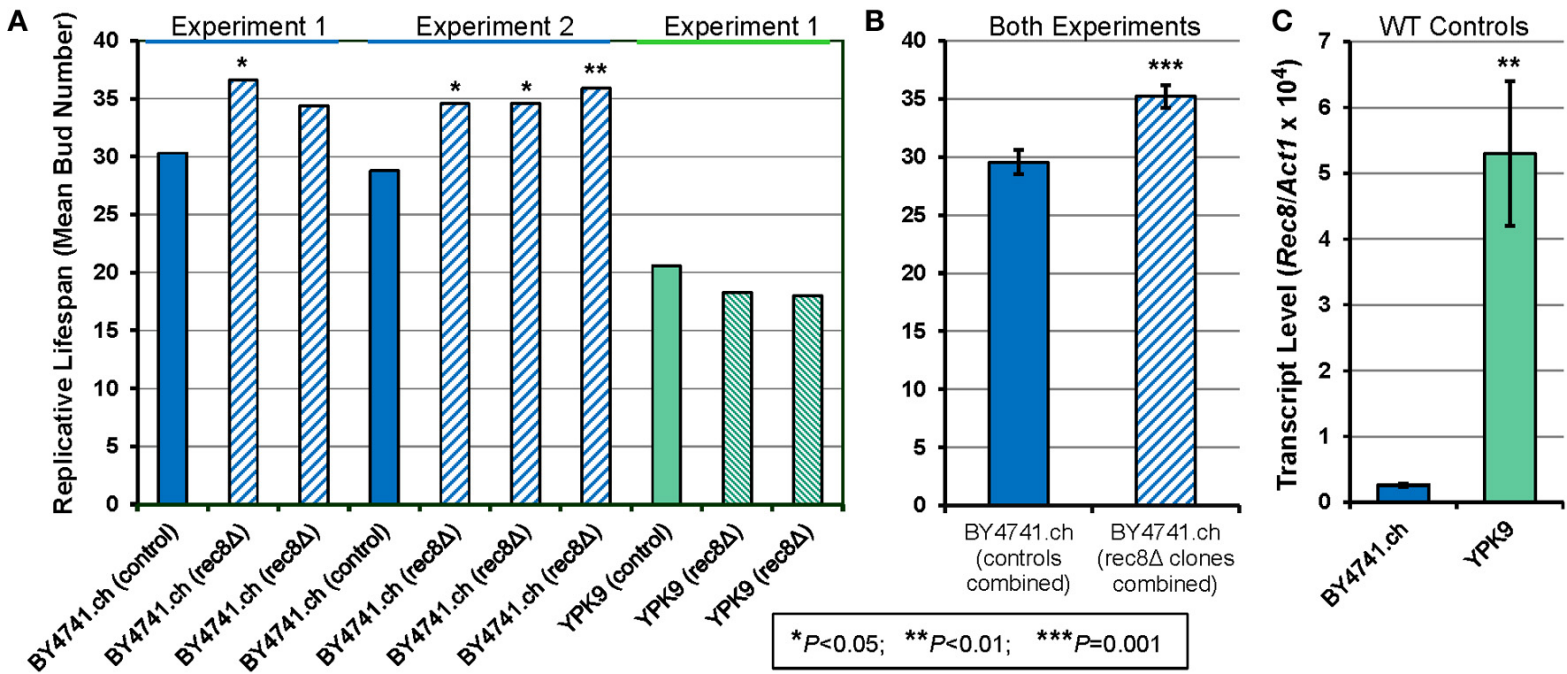
**Deletion of *REC8* increases replicative lifespan in a long-lived yeast strain that limits transcripts of this gene. (A)** Replicative lifespan was measured for the BY4741.ch strain and 5 independent deletion mutations, all assessed as haploids, in two experiments. In the first experiment, strain YPK9 and two *REC8* deletion mutants were also assessed. Means lifespans (in generations) are shown, with the significance of differences from the parental control strain indicated by asterisks. Four of the five BY4741 deletion clones were significantly longer lived than the within-experiment control, by *P* < 0.05; the combined significance of the 5 clonal life extensions was 4 × 10^−8^. **(B)** The group means and standard deviations are shown for the two genotype groups, treating each mean lifespan of a control or *rec8*Δ clone as a single point. Even by this conservative approach, the 5 *rec8*Δ clones differed from the two control assays at *P* = 0.001. **(C)** Transcript levels were assessed by quantitative real-time polymerase chain reaction (RT-PCR) for *REC8* relative to *ACT1* (actin), as described in Materials and Methods. Results are shown for triplicate cultures of the wild-type strains, with error bars indicating standard deviations. ^*^P < 0.05; ^**^P < 0.01; ^***^P = 0.001.

The Rec8p cohesin-complex proteins of fission and budding yeast are functionally analogous to the nematode REC-8 protein, and are considered to be its orthologs (http://www.wormbase.org/, Pasierbek et al., 2001). We tested the effect of *REC8* deletion on the number of mitotic progeny produced per mother cell, in the budding yeast *Saccharomyces cerevisiae*. This assay, performed as described previously (Jiang et al., 2000), was initially conducted for both the long-lived strain BY4741 (mean lifespan 29–30 bud generations) and a short-lived strain, YPK9 (20–21 bud generations). As summarized in Figure 4, replicative lifespan increased significantly in 4 of 5 of BY4741 *rec8*Δ clones, whereas it declined slightly but insignificantly in two YPK9 *rec8*Δ clones. The BY4741 *rec8*Δ clones increased in mean number of progeny, relative to their within-experiment controls, from 30.2 to 36.6 (21%, *P* < 0.03) and 34.4 (14%, not significant) in the first experiment. In a second experiment, 3 BY4741 *rec8*Δ clones gained 20, 20, and 25% in replicative potential (*P* < 0.05, 0.03, and 0.01 respectively). Combining the data for all five clones relative to their respective controls, the longevity extension upon deleting the *REC8* gene was 20 ± 4% (SD) with an overall significance of *P* < 5 × 10^−8^. We also calculated the significance (Figure 4B) by a simple *t*-test comparison treating each mean value as a single data point, which resulted in a more conservative *P*-value of 0.001. Because strain YPK9 differed markedly from BY4741, we inquired whether this might relate to the relative abundance of *REC8* transcripts in the two haploid strains (which undergo mitosis but not meiosis). *REC8* expression was 20-fold greater in YPK9 (Figure 4C), suggesting that this strain must not be under strong selection against harmful effects of inappropriate Rec8p expression in mitotic cells.

## Discussion

### Positional candidate genes for life-span effects

Confirmed and putative genes in the *lsq4* interval on chromosome IV are “positional candidates” that may underlie the traits associated with this QTL. These include 33 genes encoding known or likely proteins, and 6 sites templating small nuclear RNAs U6-8 through U6-13 (Table 2). The region contains a further 14 open reading frames (ORFs) that are unsupported either by homology to known genes or by RNA-derived clones, two of which are annotated as pseudogenes. Any of the candidate genes could be responsible for the allelic effects we demonstrated with respect to lifespan, resistance to heat and superoxide radical, and male incidence. Although different *lsq4* traits could, in principle, arise from polymorphisms in different genes, a single gene that accounts for all of the observed QTL phenotypes would provide the most parsimonious explanation.

None of the identified candidates in the *lsq4* interval were previously implicated in pathways modulating lifespan or stress resistance, although several could plausibly play such roles. They include genes encoding: (1) Four protein kinases and one phosphatase, which could be involved in signal transduction; K09B11.5 encodes a tyrosine protein kinase related to a yeast CDK and human/mouse Fer; (2) Two proteins with zinc-finger domains (e.g., MEX-5) and one with a winged-helix domain, characteristic of DNA-binding modulators of transcription; (3) The REC-8 protein, a cohesin required for meiosis and corresponding to SSC-1 in mitosis; (4) FAR-6, a fatty acid/retinol-binding protein; (5) AGT-1, an O_6_-alkyl-guanosine DNA-methyltransferase; and (6) Two RNA-binding proteins (PUF-3 and DCAP-2) that may affect mRNA stability.

### Differential effects of gene allele on transcript levels

Genomewide microarray analysis can provide clues as to the nature of the gene underlying a QTL. Of the 22 genes for which expression depended most significantly on the *lsq4* allele (each with false discovery rate *q* = 0), all were expressed at higher levels in CL2a than in SR708; 12 are members of the MSP (major sperm protein) family, and a further three are unknown proteins containing MSP-like domains (Table 1A). These results were corroborated and made more quantitative by RT-PCR targeting a region shared by many *msp* genes, or distinguishing two particular genes, *msp-142* and *msp-56* (Table 1B). For each target, CL2a showed between 4- and 5-fold more transcripts than SR708. Such a concerted shift in gene expression suggests that the key dimorphic gene within *lsq4* may encode a factor modulating a differentiation state, or the transcription or stability of mRNAs, which could then affect transcript abundances of many other genes without regard to their chromosomal locations. Of the genes situated in the *lsq4* interval, those most likely to alter transcript levels of other genes are *mex-5* (encoding a protein with two zinc-finger domains, expressed in germ-line cells of adults), Y38H8A.5 (encoding a p57^KIP2^ zinc-finger protein), Y45F10A.7 (encoding a protein with a winged-helix transcriptional repressor domain), *lsm-3* (encoding a small nuclear ribonucleoprotein), and *puf-3* (encoding an RNA-binding translational suppressor, which might affect transcript levels indirectly). Although conserved motifs (potential binding sites for proteins or RNAs, which might affect rates of transcription or mRNA degradation) were enriched with high significance in the set of genes most decisively affected by the choice of *lsq4* allele, the distribution of those motifs (Table 1, column 4) was not sufficient to explain the profile of differential gene transcription.

### Dimorphism in *rec-8* accounts for all observed *lsq4* traits

Multiple lines of evidence converge on *rec-8* as the sole gene responsible for multiple phenotypes that depend on the allele of *lsq4*. First, *rec-8* is the only gene in this interval that had been previously shown to influence male incidence: both *rec-8*-deficient mutants and wild-type worms exposed to *rec-8* dsRNA have exceptionally high male frequencies, increasing nearly 100-fold following RNAi treatment (Pasierbek et al., 2001). We observed a higher proportion of males in the CL2a strain DR1345 (1.25 ± 0.29% s.e.m.) than has been seen in other wild-type isolates such as Bristol-N2, Bergerac-BO [RW7000], or a different CL2a subline [CB4857], each of which produces 0.1–0.2% males (Hodgkin and Doniach, 1997). Male frequency in the SR708 recombinant-congenic line, in which a narrow segment of Bergerac-BO DNA is embedded in chromosome IV of CL2a, was 0.2 ± 0.1%, 6- to 7-fold less than CL2a (chi-squared *P* < 10^−5^) in each of two experiments. CL2a has 4- to 5-fold higher transcript levels of genes encoding Major Sperm Proteins (Table 1B), as well as other genes expected to be expressed chiefly in sperm (Table 1A). Thus, allelic differences in the frequency of males, due to the “HIM” phenotype associated with low REC-8 activity, would markedly affect the abundance of male sperm, and of transcripts specific to male-produced sperm (Tarr and Scott, 2004), which include most MSP-domain genes (Tarr and Scott, 2005).

Only one previous report has linked male incidence to longevity. Mutation of *him-6*, the *C. elegans* ortholog of human *BLM* (encoding the RecQ helicase that underlies Bloom-syndrome progeria), in the nematode causes genomic instability leading to increased generation of males while *reducing* lifespan (Wicky et al., 2004; Grabowski et al., 2005). Based on such observations, neither the HIM trait nor genome destabilization *per se* would have been expected to improve longevity. Mutations that reduce *C. elegans* sperm number without altering male incidence can either modestly extend lifespan, like *spe-10* (Cypser and Johnson, 1999) and *spe-26* (Kim and Sun, 2007), or shorten it like *folt-1* (Austin et al., 2010). It thus appears unlikely that an increase in total spermatogenesis could account for the greater longevity associated with the CL2a allele of *lsq4*; in fact just the opposite effect, a reduction in lifespan with increased hermaphrodite sperm production, has been described (Van Voorhies, 1992).

RNA interference has the potential to test genes for their functional roles, but it carries much stronger inferential power when a gene knockdown produces a positive effect, since detrimental effects can (and do) arise as artifacts. Of 14 *lsq4*-interval genes tested for an effect on lifespan when targeted by RNA interference, only *rec-8* produced significant and reproducible effects, extending lifespan at least 25% in two of three independent experiments (Figure 1 and Table 4). This effect size accounts for most or all of the 24–30% life extension observed for the *lsq4* interval as a whole (Vertino et al., 2011). Similarly, *rec-8* was the only tested gene for which RNAi improved survival of paraquat (which causes oxidative stress by generation of superoxide), and one of only two that enhanced thermotolerance (Figure 2). These data create a compelling argument that the longevity and stress-resistance traits of *lsq4* can be explained by differences in *rec-8* expression, arising from allelic alterations in the gene sequence. We note that QTL alleles and mutations extending lifespan are often associated with resistance to one or more (but not all) stresses (Lithgow and Walker, 2002; Shmookler Reis et al., 2007).

We analyzed the 5 kilobase-pairs upstream of *rec-8* (reading from *mex-5* to *rec-8*), but found no sequence differences between these strains. At least one single-nucleotide polymorphism (SNP), pkP4058/WBvar00240602, distinguishes between strains CL2a and SR708 in the region just downstream of *rec-8* (Vertino et al., 2011). Intronic and coding-sequence SNPs within the *rec-8* gene were found for these strains at ~0.1% of nucleotides (i.e., averaging 1 SNP per kbp). Any of these could impact expression, but a systematic mutational scan would be required to determine which SNP is responsible for the QTL differences in traits and *rec-8* transcript levels.

### REC-8 alters survival through effects on adult worms

Each of the distinctive traits associated with the CL2a allele at *lsq4* was reproduced or exceeded in recombinant-congenic strain SR708 (differing from CL2a at only a few nucleotides, spanning less than 0.33% of the genome), by exposure of those worms to *rec-8* dsRNA. Because germline cells begin to proliferate during the first larval (L1) stage, with germ-cell numbers amplifying progressively in L2–L4 larvae and young adults, RNAi treatment for male-incidence counts was initiated prior to embryogenesis of the tested parents, and maintained throughout their development and egg-laying. For other traits, however, we were able to vary the period of RNAi exposure, commencing either prior to oocyte maturation or at the onset of adulthood. Exposure of strain SR708 to *rec-8* dsRNA only during adulthood extended adult lifespan by 26%, which was not inferior to treatment throughout development and aging (Figure 1 and Table 4). This implies that the survival benefits of reduced *rec-8* expression primarily reflect diminished REC-8 activity in adults, with little or no involvement of differential effects during development. We cannot exclude a mechanism that entails altered *rec-8* expression in germ cells (see the last section of Discussion), but note that this mode of action must be restricted to the germ cells of adult worms, since RNAi treatment was equally effective whether begun at the L4/adult molt or prior to larval development, when most germ cell proliferation occurs.

### Deletion of *REC8* can extend the replicative lifespan of haploid budding yeast

*REC8* deletion from a long-lived *S. cerevisiae* strain, BY4741, reproducibly added about 20% to the replicative life span of haploid cultures. This was true whether the yeast were grown on glucose or raffinose, and on either carbon source it also applied to rho° mutants of BY4741 (data not shown). We are aware of only one previous study to assess effects of *REC8* deletion on *S. cerevisiae* survival, which reported *rec8*Δ in an S288C background to have improved survival after “hibernation,” i.e., 58 months storage at 4°C (Postma et al., 2009). Why, then, was mitotic survival not increased (and perhaps reduced, although insignificantly) by the same deletion in the YPK9 strain? *REC8* expression in mitotic yeast cells (in particular the haploid cultures studied here, since they do not normally undergo meiosis) is ectopic expression of a meiotic gene. We conjecture that such “leaky” and inappropriate expression is harmful because it aggravates the normal age-dependent destabilization of their genome (McMurray and Gottschling, 2004; Hachinohe et al., 2011). In this case, the YPK9 strain may owe its brief lifespan (30% less than BY4741; see Figure 4) to genomic instability from other sources, which overshadow any contribution from Rec8p, or to causes unrelated to genomic aberrations, leading to death before genome instability can play a role. The *REC8* transcript level was roughly 20-fold higher (relative to actin mRNA) in the wild-type YPK9 strain than in BY4741.ch (Figure 4C), consistent with the absence of any selection against high *REC8* expression in YPK9.

### Possible mechanisms and evolutionary implications

Since *rec-8* was not previously recognized as a life-assurance gene or LAG (Tacutu et al., 2013; http://genomics.senescence.info/genes/index.html), nor is it known to interact with any nematode LAGs (Tacutu et al., 2010, 2012; http://netage-project.org/), it appears to employ a novel pathway not hitherto recognized to impact longevity. We infer that this pathway has been widely conserved, based on its effects in both nematodes and yeast. Indeed, *rec8* was one of 6 genes with evidence of strong positive selection in the bowhead whale (lifespan >200 years), after its divergence from the Minke whale (lifespan <50 years). It is of interest that the 5 other genes are involved in cancer and/or stress responses (de Magalhaes, Pers. Commun.).

On the basis of transcript profiles, the HIM (high incidence of males) trait, and RNAi results, dimorphic expression of *rec-8* appears to be a sufficient explanation for all observed *lsq4*-associated traits. The molecular basis for those *rec-8* traits remains unresolved. Why should lower *rec-8* expression or activity be associated with greater longevity and resistance to stresses? Although reduction in REC-8 protein destabilizes the germ-line genome (generating males by X-chromosome non-disjunction), it might *favor stability* of somatic genomes if normal transcription levels permit “leaky” expression in soma, causing as-yet-unknown harmful effects. Despite evidence from immunocytology that the REC-8 cohesin is observed only in gonads (WormBase, www.wormbase.org), low-level expression in other tissues has not been excluded. Such ectopic expression could destabilize the somatic genome throughout life, potentially reducing lifespan—as occurs in *C. elegans* when a *him-6* mutation produces genome instability (Grabowski et al., 2005). REC-8 has no known role in non-meiotic cells, but its ectopic presence in somatic cells might increase the potential for chromosome fragmentation during aging, consistent with the well-established association of DNA-repair gene expression with aging (Lee et al., 1999; Park et al., 1999; Doria et al., 2004; McCarroll et al., 2004) and reduced induction of such genes in very long-lived mutants (Ayyadevara et al., 2009). The fission yeast (*S. pombe*) ortholog of REC-8 protein, Rec8p, is also strongly induced in meiosis, but low-level expression in vegetative (mitotic) cells was unambiguously demonstrated for 3 epitope-tagged *rec8* constructs (Krawchuck and Wahls, Pers. Commun.). An alternative hypothesis, which does not invoke REC-8 expression in somatic cells, is that genotoxicity in adult germ cells elicits a hermetic protective response in the soma. This possibility was raised by a recent report that DNA damage in germ cells elicits systemic stress resistance through an innate-immunity response (Ermolaeva et al., 2013). Although effects on lifespan were not demonstrated, they might be anticipated as a consequence of altered response to multiple stresses.

Nematode resistance to several stressors could arise from *rec-8* suppression under either hypothesis. Thermal and oxidative stresses may in effect “add insult to injury” by increasing the normal burden on DNA repair machinery, which is exacerbated by somatic expression of *rec-8*. It is a common observation in nematode laboratories that male incidence (and thus X-chromosome non-disjunction) is increased after heat shock or other transient stresses (Ebert et al., 1993; Morran et al., 2009).

We note that *rec-8* polymorphism, in our proposed model, constitutes an example of antagonistic pleiotropy in which the allele that is usually preferred under natural selection (i.e., the allele conferring higher *rec-8* expression, because it helps to maintain the very low incidence of males that predominates in wild-derived strains), at the same time leads to a disadvantageous phenotype during aging of somatic tissues (shorter lifespan and reduced resistance to potentially genotoxic stresses). Antagonistic pleiotropy with respect to lifespan is thought to arise because long-term survival, and even reproduction later in life, are subject to a progressively declining force of natural selection (Kirkwood and Rose, 1991; Mueller and Rose, 1996). Dimorphic effects of *rec-8* may also exemplify a special subclass of balanced polymorphisms wherein each allele confers a selective advantage in a particular context (Modiano et al., 2008; Seidel et al., 2008; Ramalho et al., 2010), since high male frequency (and hence increased mating) constitutes wasted reproductive effort under benign conditions but provides increased segregation of novel gene combinations, and hence greater opportunity for evolutionary adaptation, under harsh conditions in which the force of selection is high.

## Funding

This work was supported by grants from the National Institute on Aging (National Institutes of Health), R01-AG091413 and P01-AG20641 to Robert J. Shmookler Reis, and R37-AG006168 to S. Michal Jazwinski. Additional support to Robert J. Shmookler Reis was provided by the Dept. of Veteran Affairs, including a Senior Research Career Scientist award; to Srinivas Ayyadevara through a pilot grant from the UAMS Pepper Center (NIH grant AG028718); and to Robert J. Shmookler Reis and Srinivas Ayyadevara from the Life Extension Foundation.

### Conflict of interest statement

The authors declare that the research was conducted in the absence of any commercial or financial relationships that could be construed as a potential conflict of interest.

## Author Note

Because Srinivas Ayyadevara and Robert J. Shmookler Reis are employees of the Veterans Administration, this article is considered to be in the public domain; its contents, however, do not represent the views of the US government or any agency thereof.

## Abbreviations

BO: *C. elegans* var. Bergerac-BO (here, strain RW7000 from R. Waterston)
cDNA: complementary DNA
CL2a: *C. elegans* var. Cl2a (here strain DR1345 from D. Riddle)
dsRNA: double-stranded RNA (produced via bidirectional T7 polymerase transcription)
HIM: high incidence of males
LS: lifespan
MLS: maximum lifespan (i.e., the lifespan of the last-surviving worm in a cohort)
MSP: major sperm protein
N2: *C. elegans* var. Bristol-N2 (here, subline N2-DRM from D. Riddle)
NC: no change
NGM: nematode growth medium
NS: not significant
PCR: polymerase chain reaction
QTL: quantitative trait locus
RT-PCR: real-time, reverse-transcriptase polymerase chain reaction
RNAi: RNA interference
SAM: Significance Analysis of Microarrays
SD: standard deviation
SEM: standard error of the mean
SNP: single nucleotide polymorphism
UV: ultraviolet.
